# m5U-SVM: identification of RNA 5-methyluridine modification sites based on multi-view features of physicochemical features and distributed representation

**DOI:** 10.1186/s12915-023-01596-0

**Published:** 2023-04-24

**Authors:** Chunyan Ao, Xiucai Ye, Tetsuya Sakurai, Quan Zou, Liang Yu

**Affiliations:** 1 School of Computer Science and Technology, Xidian University, Xi’an, China; 2 Department of Computer Science, University of Tsukuba, Tsukuba, Japan; 3 Institute of Fundamental and Frontier Sciences, University of Electronic Science and Technology of China, Chengdu, China; 4 Yangtze Delta Region Institute (Quzhou), University of Electronic Science and Technology of China, Quzhou, China

**Keywords:** 5-Methyluridine, Support vector machines, Multi-view feature, Word2Vec

## Abstract

**Background:**

RNA 5-methyluridine (m5U) modifications are obtained by methylation at the C_5_ position of uridine catalyzed by pyrimidine methylation transferase, which is related to the development of human diseases. Accurate identification of m5U modification sites from RNA sequences can contribute to the understanding of their biological functions and the pathogenesis of related diseases. Compared to traditional experimental methods, computational methods developed based on machine learning with ease of use can identify modification sites from RNA sequences in an efficient and time-saving manner. Despite the good performance of these computational methods, there are some drawbacks and limitations.

**Results:**

In this study, we have developed a novel predictor, m5U-SVM, based on multi-view features and machine learning algorithms to construct predictive models for identifying m5U modification sites from RNA sequences. In this method, we used four traditional physicochemical features and distributed representation features. The optimized multi-view features were obtained from the four fused traditional physicochemical features by using the two-step LightGBM and IFS methods, and then the distributed representation features were fused with the optimized physicochemical features to obtain the new multi-view features. The best performing classifier, support vector machine, was identified by screening different machine learning algorithms. Compared with the results, the performance of the proposed model is better than that of the existing state-of-the-art tool.

**Conclusions:**

m5U-SVM provides an effective tool that successfully captures sequence-related attributes of modifications and can accurately predict m5U modification sites from RNA sequences. The identification of m5U modification sites helps to understand and delve into the related biological processes and functions.

**Supplementary Information:**

The online version contains supplementary material available at 10.1186/s12915-023-01596-0.

## Background

Posttranscriptional modification of RNA, the processing of primary transcribed RNA into mature RNA in organisms, is an important component of the field of gene regulation. Such modifications refer to the introduction of chemical groups to RNA bases [1, 2]. As a result of rapid advances in genomics and molecular biology, over 170 posttranscriptional modifications have been identified using high-throughput techniques and related experiments. RNA posttranscriptional modification, a type of regulation that occurs at the posttranscriptional level, not only enriches genetic information but also plays crucial roles in multiple biological processes, such as RNA localization and degradation [3], dynamic changes in RNA structure [4], and RNA splicing [5]. In addition, due to abnormal expression levels of RNA methyltransferases and de-methyltransferases, multiple types of RNA modifications are closely associated with the development of human disease and gene expression [6]. RNA posttranscriptional modifications include N6-methyladenosine (m6A), N7-methylguanosine (m7G), 2′-O-methyladenosine (Am), 5-methylcytidine (m5C), N1-methyladenosine (m1A), 2′-O-methylguanosine (Gm), and 5-methyluridine (m5U) [7]. 5-Methyluridine (m5U) is a post-transcriptional modification of RNA. The m5U modifications are pyrimidine modifications, which are obtained by methylation at the C_5_ position of uridine catalyzed by pyrimidine methylation transferase [8]. The enzymes that catalyze the m5U modification are different for different species: TRMT2A and TRMT2B in mammals [9, 10], Trm2p in *S. cerevisiae* [11], and TrmA in *E. coli* [12].

Research has shown that aberrant expression of m5U-modified ribonucleosides is related to the development of human diseases such as systemic lupus erythematosus and breast cancer [13, 14]. Therefore, studies on the identification of m5U modification sites can contribute to the understanding and in-depth study of the associated biological processes and functions. Compared to other well-studied modifications, there is a paucity of studies on the identification and functional characterization of m5U modification sites. For m5U modification site identification, several wet experimental methods have been developed, including fluorouracil-induced catalytic crosslinking sequencing [15]. However, this class of methods is costly and time-consuming. To improve on these limitations, researchers have developed computational methods to identify m5U modification sites. At present, there are few computational methods to identify m5U-modified sites [16, 17]. Among them, Jiang et al. [17] developed the identification method m5UPred for human m5U-modified site data using the support vector machine (SVM) algorithm and based on sequence-derived information features (nucleotide density and nucleotide chemistry). Although the computational method achieved an AUC of over 0.954 under fivefold cross-validation and an independent test set, the dataset used by this method was not redundantly processed, and the performance of the model caused overfitting.

Therefore, a new prediction tool, m5U-SVM, is developed to address the shortcomings of current prediction tools. In this research, we used a collocation of traditional physicochemical features and distributed representation features to construct a prediction model based on multi-view features and SVM. First, the optimized multi-view physicochemical features were filtered by fusing the four traditional physicochemical features using the two-step method of LightGBM and IFS. Then, the new multi-view features were obtained by stitching the embedded features with the optimized physicochemical features. Finally, the prediction models were constructed by screening with different machine learning algorithms using SVM and multi-view features. Under tenfold cross-validation (tenfold CV), the prediction models had prediction accuracies of 88.876% and 94.358% for full transcript and mature mRNA modes, respectively; the prediction accuracies of the independent testing were 90.821% and 94.106%. In addition, Shapley Additive explanation (SHAP) was used to interpret the model predictions.

## Methods

**Fig. 1:**
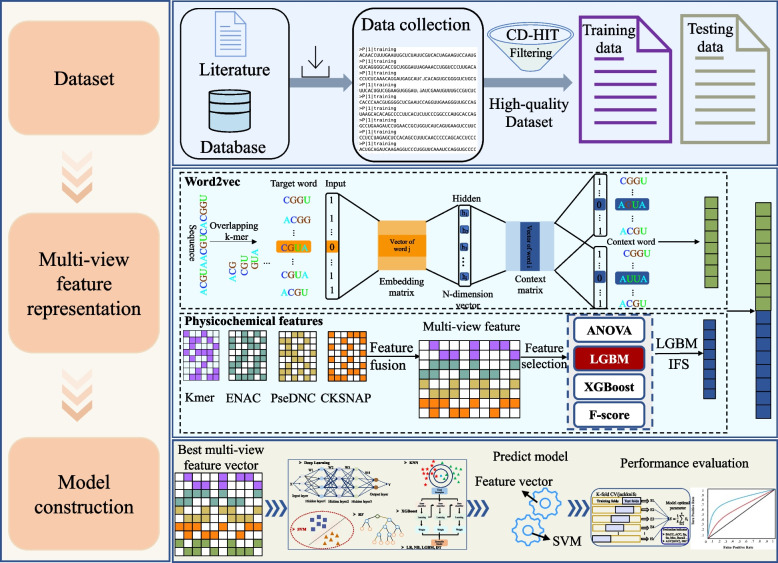
Overall architecture of the prediction model

In this work, we propose a new predictor, m5U-SVM, whose overall construction workflow is shown in Fig. 1. It consists of three main parts: (a) dataset collection and pre-processing, (b) multi-view feature representation, and (c) model training and performance evaluation. A detailed description of each part follows.

### Dataset collection and pre-processing

The original datasets of RNA m5U modification sites were derived from Jiang et al. [17]. The positive sample was processed to obtain a 41 nt modified sequence, and the modified U site in the middle of the sequence was determined experimentally. Negative samples were obtained by randomly selecting unmodified uridine sites from the same transcripts as positive samples. The full transcript mode dataset obtained consisted of 3696 positive samples and 3696 negative samples, and the mature mRNA mode dataset is consisted of 1232 positive samples and 1232 negative samples. Homologous sequences were not removed from the acquired sequences. The performance of the model would be affected if the sequence homology was too high. Therefore, we used CD-HIT [18] to remove homology from the obtained full transcript mode m5U modification site data, with a threshold of 80%. Finally, the m5U modification site dataset contained 1534/2862 full transcript mode training sets, 500/731 full transcript mode independent test sets, 983/985 mature mRNA mode training sets, and 245/247 mature mRNA mode independent test sets. The relevant datasets can be accessed at https://github.com/aochunyan/m5U-SVM/tree/main/Dataset.

### Feature extraction

Feature extraction is the process of transforming raw data that cannot be recognized by machine learning algorithms into data features that can be recognized. In this study, multi-view feature representation methods are used to convert nucleotide sequences into feature vectors, which contain two major classes of feature representation methods, namely, physicochemical feature representation methods and distributed representation methods.

(I) Physicochemical feature representation

Adopting an effective feature-encoding method to transform nucleotide sequences represented by strings into feature vectors represented by values is a key part of machine learning classification models, which directly affects the performance of the model. In this research, four physicochemical feature encoding strategies were employed to formulate the RNA modification fragments. These features are enhanced nucleic acid composition (ENAC), the composition of k-spaced nucleic acid pairs (CKSNAP), Kmer, and pseudo dinucleotide composition (PseDNC). These encoding methods all employ the iLearn and iLearnplus toolkits [19, 20].

*ENAC*: Nucleotide composition (NC) (i.e., Kmer nucleotide frequency) is a classic coding method for expressing the features of a nucleotide sequence, which is used to calculate the frequency of occurrence for each Kmer nucleotide in the sample sequence and will generate a 4^ K^-dimensional feature vector. ENAC is a variation method 1-mer nucleic acid frequency (i.e., NAC). ENAC figures NAC based on a fixed-length sequence window that slides from the 5′ to 3′ terminus of each RNA sequence in succession, in which the length of the window is set to 5 [21]. Moreover, the following formula is used to calculate Kmer nucleotide frequency:

1 $$f\left({n}_{1}{n}_{2}\dots {n}_{K}\right)=\frac{N\left({n}_{1}{n}_{2}\dots {n}_{K}\right)}{\left(L-K+1\right)},\left({n}_{k}\in \left(A, G,C,U\right)\right)$$

where ${n}_{1}{n}_{2}\dots {n}_{K}$ indicates a Kmer nucleotide component, and $N\left({n}_{1}{n}_{2}\dots {n}_{K}\right)$ is the number of occurrences of ${n}_{1}{n}_{2}\dots {n}_{i}\dots {n}_{K}$ in an RNA sequence. ENAC is adopted to calculate the frequency of occurrence of each nucleotide in the sequence to generate a ${4}^{k}$ feature vector.

*CKSNAP*: The CKSNAP feature encoding scheme calculates the frequency of nucleic acid pairs separated by any *k* nucleic acids (*k* = 0, 1, 2,…, 5) [22]. Taking *k* = 0 as an example, there are 16 0-spaced nucleic acid pairs (*i.e.*, “AA,” “AC,” “AG,” “AU,” “CA,” “CC,” “CG,” “CU,” “GA,” “GC,” “GG,” “GU,” “UA,” “UC,” “UG,” and “UU”). Subsequently, a feature vector can be defined as:

2 $${\left(\frac{{\mathrm{N}}_{AA}}{{N}_{total}},\frac{{N}_{AC}}{{N}_{total}},\frac{{N}_{AG}}{{N}_{total}},\dots ,\frac{{N}_{UU}}{{N}_{total}}\right)}_{16}$$

The value of each descriptor denotes the composition of the corresponding nucleic acid pair in the nucleotide sequence. The CKSNAP feature-encoding strategy is used to calculate the frequency of occurrence of nucleotides separated by an arbitrary number (*k*) of nucleotides, with *k* taking values in the range of 0–6 {}^20^]. For example, if the nucleic acid pair AA appears *m* times in the nucleotide sequence, the composition of nucleic acid pair AA is equal to m divided by the total number of 0-spaced nucleic acid pairs (${N}_{total}$) in the nucleotide sequence. In this study, we set *k* = 0, 1, 2, 3, 4, and 5, the corresponding value of ${N}_{total}$ is L–1, L–2, L–3, L–4, L–5, and L–6 for a sequence of length *L*, respectively.

*Kmer*: Kmer is the most direct approach to represent the RNA sequences, which are defined as the occurrence frequencies of k-neighboring nucleic acids [20, 23]. For example, the Kmer (*k* = 3) descriptor was calculated as follows:

3 $${V}_{Kmer}=\frac{{N}_{t}}{N} t\in (AAA, AAC,\dots \dots , UGC)$$

where ${N}_{t}$ is the number of Kmer type *t*, while *N* is the length of a nucleotide sequence.

*PseDNC*: PseDNC is a feature extraction algorithm commonly used in the pseudo nucleic acid composition (PseNAC) algorithm, which is used to mine the sequence information and effects of RNA modification sequences [24]. PseDNC feature encoding can cover local and global sequence-order from the given sequence. It is defined as follows:

4 $$P={\left({p}_{1},{p}_{2},\dots ,{p}_{16},{p}_{16+1},\dots ,{p}_{16+\lambda }\right)}^{T}$$

5 $${p}_{k}=\left\{\begin{array}{c}\frac{{f}_{k}}{{\sum }_{i=1}^{16}{f}_{i}+w{\sum }_{j=1}^{\lambda }{\theta }_{j}},\left(1\le \kappa \le 16\right)\\ \frac{w{\theta }_{k-16}}{{\sum }_{i=1}^{16}{f}_{i}+w{\sum }_{j=1}^{\lambda }{\theta }_{j}},\left(17\le \kappa \le 16+\lambda \right)\end{array}\right.$$

where ${f}_{k} (k=\mathrm{1,2},..., 16)$ reflects normalized dinucleotide frequency in the given sequence, $\lambda$ represents the highest counted rank of the correlation along the given sequence, $w\left(0-1\right)$ is the weight factor, and ${\theta }_{j}(j=\mathrm{1,2},\dots ,\lambda )$ is the $j-$ tier correlation factor, which is defined as:

6 $$\left\{\begin{array}{c}\theta_{1}=\frac1{L-2}{\textstyle\sum\nolimits_{i=1}^{L-2}}\Theta\left(R_iR_{i+1},R_{i+1}R_{i+2}\right) \\...\\ \theta_\lambda=\frac1{L-1-\lambda}{\textstyle\sum\nolimits_{i=a}^{L-1-\lambda}}\Theta\left(R_iR_{i+1},R_{i+\lambda}R_{i+\lambda+1}\right)\end{array}\right.$$

where the correlation function is defined as

7 $$\Theta \left({R}_{i}{R}_{i+1},{R}_{j}{R}_{j+1}\right)=\frac{1}{\mu }\sum\nolimits_{\mu =1}^{\mu }{\left({C}_{u}\left({R}_{i}{R}_{i+1}\right)-{C}_{u}\left({R}_{j}{R}_{j+1}\right)\right)}^{2}$$

where $\mu$ denotes the number of physicochemical indexes. Six physicochemical indexes, including rise, roll, shift, slide, tilt and twist, were considered in this work. ${C}_{u}\left({R}_{i}{R}_{i+1}\right)$ is the numerical value of the $uth$ physicochemical index of the dinucleotide ${R}_{i}{R}_{i+1}$ at position $i$, and ${C}_{u}\left({R}_{j}{R}_{j+1}\right)$ denotes the corresponding value of the dinucleotide ${R}_{j}{R}_{j+1}$ at position $j$.

(II) Word2Vec feature representation

Distributed representation methods were originally developed as an encoding method for the field of natural language processing, which included two embedding methods, word2vec [25] and doc2vec [26], to obtain a distributed representation of words and documents. Inspired by natural language processing distributed representation methods, researchers have applied the word2vec model in biological sequence recognition studies [27, 28], where sample sequences and bases correspond to sentences and words in natural language. In word2vec, there are two main neural networks in which weights to learn the context of words are used to generate word embedding vectors: continuous bag-of-words (CBOW) and continuous skip-gram (skip-gram). In this study, we used the skip-gram algorithm of the word2vec model for distributed representation of modification site sequences. This embedding method learns the co-occurrence statistics and distributed representation of the Kmer by projecting it onto an n-dimensional space *D*^*n*^. For a brief description of the encoding process of the embedding method word2vec model, the sequence of modifier sites of length $L$ is considered as a sentence, and the sequence is first partitioned into Kmer subsequences using a sliding window size of $k$, where the subsequences of all sequences are obtained with a step size of 1, ultimately generating a vocabulary $W$ of biological sequences, i.e., a biological corpus. Based on the corresponding indices of the corpus $W$, the surrounding words are then predicted from the current word using the skip-gram algorithm in the word2vec model, and then the vector of words in each subsequence is queried from the embedding matrix. Finally, each sequence is represented as a vector of $S$ by the average of all corpora in the sequence.

### Feature optimization

For the four physicochemical property descriptions of the feature extraction methods, the number of feature dimensions obtained is determined by the relevant feature extraction method hyperparameters. Therefore, the hyperparameters of the four feature descriptors were optimized to obtain the physicochemical features with optimal results, and the hyperparameters optimization ranges and results for each feature method are shown in Additional file 1: Table S1. Feature selection can play a crucial role in improving the performance of prediction models. In this research, four different physicochemical property features are combined, which can have high-dimensional features and cause information redundancy, which can affect the performance of the model and increase the computational complexity. Therefore, a two-step feature optimization approach was used to select the optimal feature subset, first selecting feature importance ranking methods such as ANOVA [29], *F*-score, LightGBM [30], and XGBoost [31] for feature ranking, and then using an accuracy-based incremental feature selection (IFS) strategy to determine the optimal feature subset.

### Model construction and performance evaluation

SVM is a generalized linear classifier to classify data in a supervised learning manner that was proposed and developed by Vapnik et al. [32]. SVM accurately classifies samples by generating the most hyperplanes to separate data points in the space using quadratic programming on the training data. In recent years, SVM has been widely used in machine learning and has achieved excellent results in machine learning tasks and bioinformatics research [33, 34]. SVM can be used to improve prediction performance using a variety of kernel methods, including Gaussian radial basis functions (RBF), sigmoid kernels, linear kernels, polynomial kernels, and more for nonlinear classification, and it performs well with small samples and nonlinear studies. Assuming a given training set of *S* = $\left\{\left({x}_{1}, {y}_{1}\right),\left({x}_{2}, {y}_{2}\right),\left({x}_{3}, {y}_{3}\right), \dots ,\left.\left({x}_{n}, {y}_{n}\right)\right\}\right.$, the data are fitted by mapping the input training set to a high-dimensional space using the nonlinear function $f(x)$ with the following equation [35]:

8 $$q\left(x\right)=\omega \cdot f\left(x\right)+v$$

where $\omega$ denotes the weights and $v$ denotes the threshold. The optimal objective function is as follows:

9 $$\underset{{}_{(\omega,\xi_i,\xi_i^\ast)}{}}{{}^{\min R}{}}=\frac12{\Arrowvert\omega\Arrowvert}^2+\sum\nolimits_{i=1}^t\left(\xi_i+\xi_i^\ast\right)\cdot C$$

The constraints are as follows:

10 $$s.t.\left\{\begin{array}{c}\begin{array}{c}q\left({x}_{i}\right)-{y}_{i}\le \varepsilon +{\xi }_{i}\\ {y}_{i}-q({x}_{i})\le \varepsilon +{\xi }_{i}^{*}\end{array}\\ {\xi }_{i}\ge 0,{\xi }_{i}^{*}\ge 0, i=\mathrm{1,2},\dots ,n\end{array}\right.$$

where both ${\xi }_{i}$ and ${\xi }_{i}^{*}$ denote relaxation variables. Both are greater than 0 when there is a prediction error, and conversely, both are equal to 0.$C$ is the penalty parameter used to reduce the fitting error.

In addition, the Lagrangian operator was introduced to address the pairwise optimization problem, calculated as follows:

11 $$L=\frac12{\Arrowvert\omega\Arrowvert}^2+C\sum\nolimits_{i=1}^n\left(\xi+\xi_i^\ast\right)-\sum\nolimits_{i=1}^n\left(u_i^\ast\xi_i^\ast+u_i\xi_i\right)+\sum\nolimits_{i=1}^n\alpha_i(q\left(x_i\right)-y_i-\varepsilon-\xi_i)+\sum\nolimits_{i=1}^n\alpha_i^\ast(y_i-q\left(x_i\right)-\varepsilon-\xi_i^\ast)$$

where ${u}_{i},{u}_{i}^{*}{,\alpha }_{i},{\alpha }_{i}^{*}$ are the Lagrangian constants. $\varepsilon$ is the allowable error. Derivation of Eq. (4), together with the introduction of the kernel function, gives a nonlinear fit function as follows:

12 $$q\left(x\right)={\sum }_{i=1}^{n}\left({\alpha }_{i}^{*}-{\alpha }_{i}\right)K(x, {x}_{i})+v$$

The above is the SVM calculation process. Before using an SVM to build a good prediction model, the following parameters should be optimized: the kernel width parameter *g*, the kernel parameter *γ*, and the regularization parameter *C*.

In this study, we used SVM to construct the prediction model and used grid search and tenfold CV to determine the optimal hyperparameters of the classifier. SVM implementation was performed using the Python package scikit-learn. The k-fold cross-validation is a common accuracy testing method in classification studies, mainly dividing the dataset into ten parts and then using each part as the validation set and the others as the training set for training and validation. The mean of the results of the ten times is used as an estimate of the accuracy of the algorithm. In addition, five commonly used evaluation metrics were chosen to assess the performance of the model, namely, sensitivity (Sn), specificity (Sp), Matthew’s correlation coefficient (MCC), accuracy (Acc), precision, and F1-score (F1), and the specific calculation of each evaluation metric is shown in the in the Additional file 5.

## Results and discussion

### Nucleotide composition analysis

**Fig. 2:**
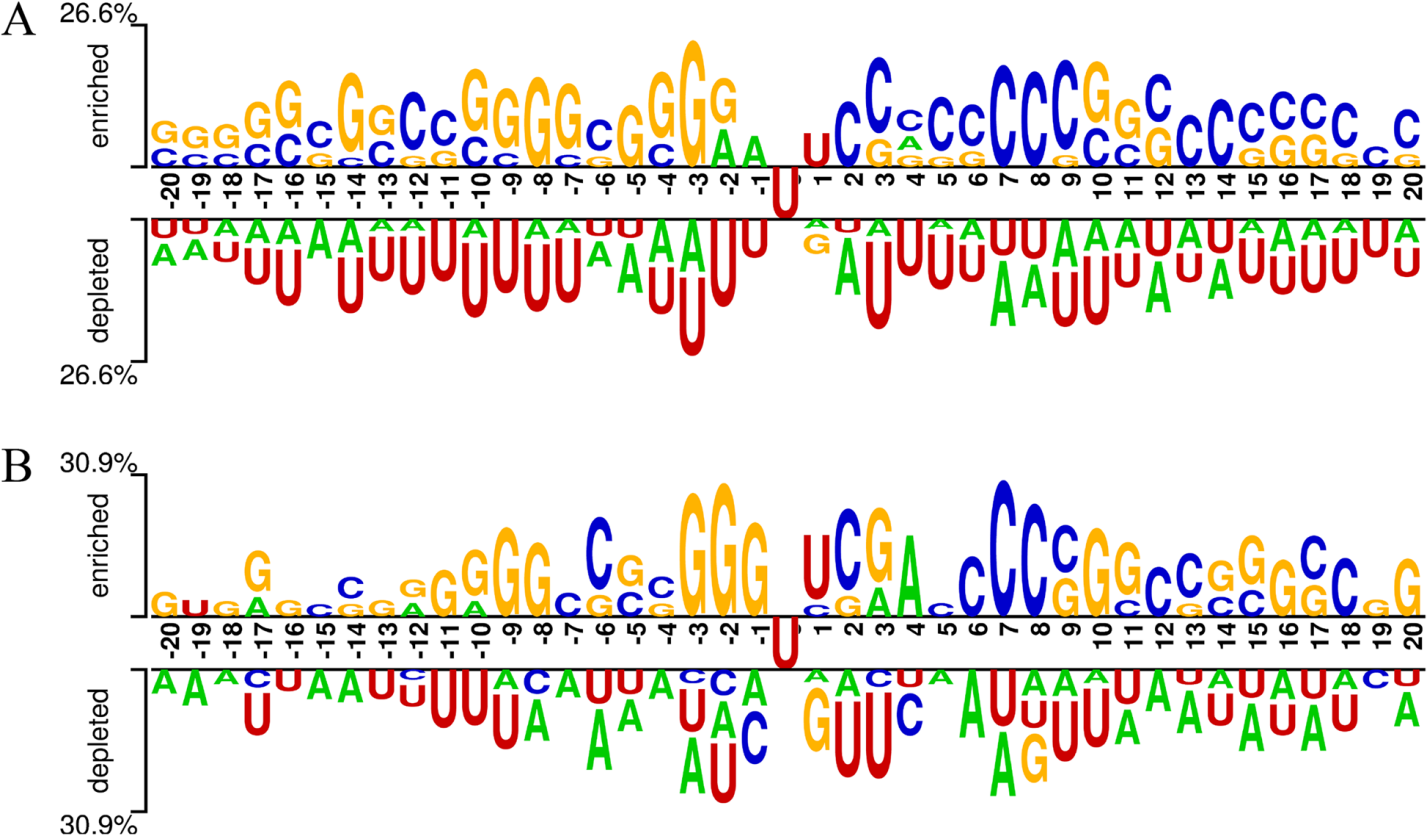
Nucleotide composition analysis. The panel above position 0 represents the m5U sites, and the panel below represents the non-m5U sites

To investigate whether the nucleotides containing m5U modification sites have a compositional deviation, the Two-Sample Logo software [36] was used to calculate the difference between sequences containing m5U modification sites and sequences without m5U modification sites, thus generating two-sample logos to visualize significantly enriched or reduced residues in m5U modification fragments. The nucleotides surrounding the m5U modification sites were statistically significant (*t*-test, *p* < 0.05), as shown in Fig. 2. Fig. 2A depicts the full transcript mode sequences (m5U and non-m5U), and Fig. 2B depicts the mature mRNA mode sequences (m5U and non-m5U). Conserved consensus motifs UUC located at positions 0 to 2 were found in both the full transcript and mature mRNA modes. At the same time, position-specific nucleotide enrichment was also found on the m5U modification site sequence. For example, some position-specific nucleotide enrichment occurred in two different modes of sequences, with G enriched at positions − 8 and − 3, C enriched at positions 7 and 8, and U enriched at position 1. There are also three contiguous G nucleotides at positions − 10, − 9, and − 8 and four contiguous C nucleotides at positions 6, 7, 8, and 9. In addition, deviations in nucleotide composition were found for different types of sequences, e.g., at position 19, the full transcript mode modification site was enriched for C, and the mature mRNA modification site was enriched for G. Similarly, at position − 1, the full transcript mode was enriched for A, and the mature mRNA mode was enriched for G. Analysis of nucleotide deviations showed that the sequence information was effective in identifying RNA m5U modification sites.

### Performance evaluation of different feature descriptors

**Fig. 3:**
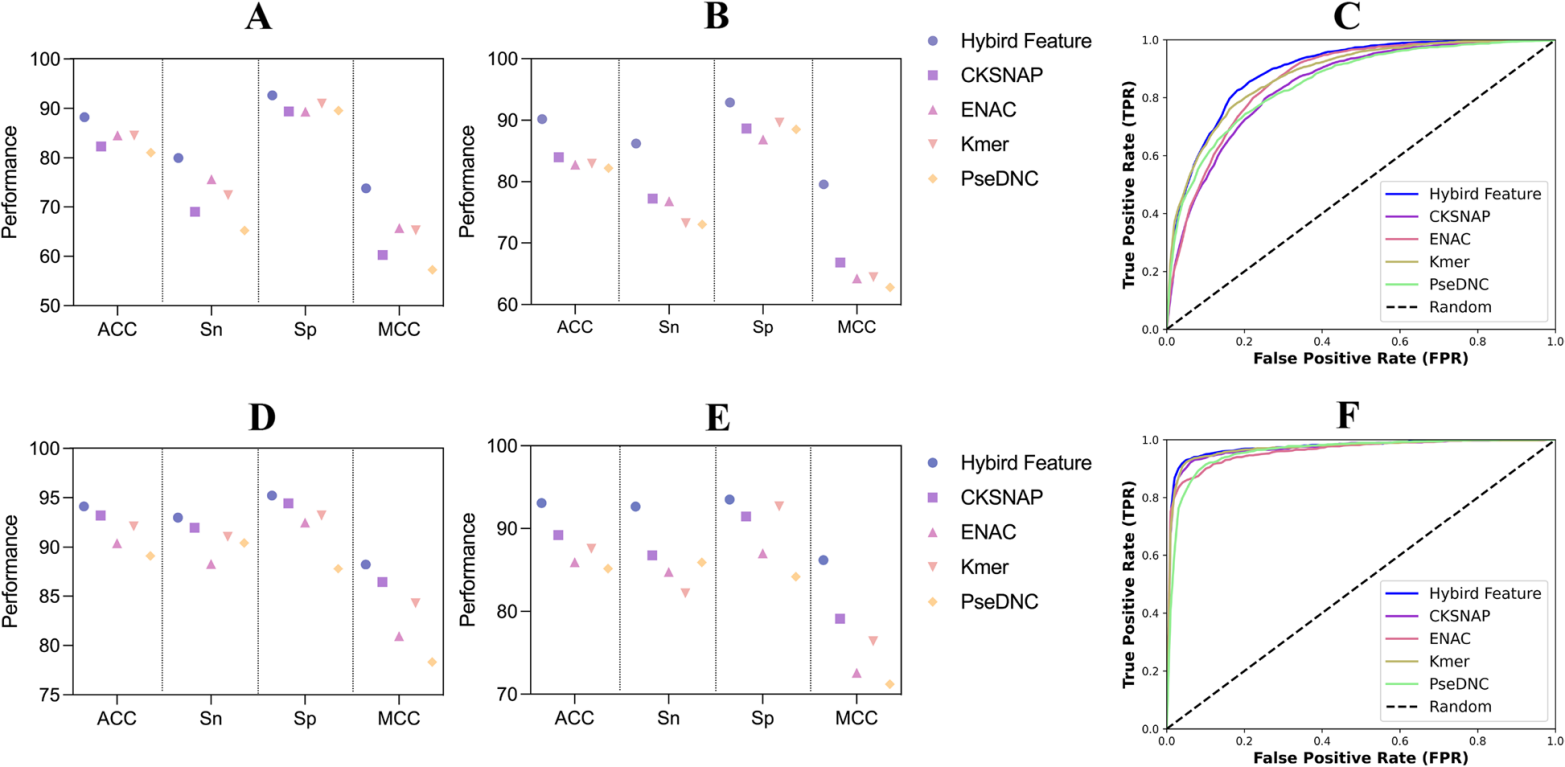
Performance comparison of different feature descriptors for full transcript and mature mRNA modes. **A** and **D** show the performance evaluations of the full transcript and mature RNA modes under tenfold CV, respectively; **B** and **E** show the performance evaluations of the full transcript and mature mRNA modes under the independent test set, respectively; **C** and **F** are the ROC curves of full transcript and mature mRNA modes under tenfold CV, respectively

In the feature extraction method section, four physicochemical feature representation methods are used, and the hyperparameters of the feature algorithm determine the feature dimensions of different features. To mine the modification sequence information as much as possible, the hyperparameters of the four feature extraction methods were optimized, and the optimal hyperparameters and the optimization results are provided in Additional file 1: Table S1. The optimization of the feature extraction method hyperparameters was performed on the full transcript mode training dataset, and the optimal hyperparameters were applied to the mature mRNA mode modification sequences. The performance of different feature representation methods for full transcript and mature mRNA modes are shown in Additional file 1: Table S2 and Fig. 3. In addition, feature fusion of the four optimized features was performed to obtain multi-view features, which is a feature vector containing four physicochemical properties. The experimental results suggest that the accuracy of the multi-view features obtained from the fusion of the four optimized features is better than that of the four single feature descriptors, with accuracies of 88.217% and 94.106% for the modified sites of full transcript and mature mRNA modes, respectively. Similarly, the other evaluation metrics were also better for multi-view features than for single-view features. For either mode of modification site under the independent test set, consistent with the results under tenfold CV, the multi-view features outperformed the single feature descriptors.

### Optimal feature selection and analysis

In this research, four physicochemical feature descriptors were fused to obtain the multi-view physicochemical features, CKSNAP + ENAC + Kmer + PseDNC (497D). The obtained features can suffer from defects such as high dimensionality and redundant information, which can affect the performance of the model. To mine the useful feature information, a two-step feature optimization method approach was chosen to perform feature optimization. First, four methods were chosen, namely, analysis of variance (ANOVA), *F*-score, LightGBM, and XGBoost, to rank the features according to their importance values, followed by IFS to determine the best multi-view physicochemical features. After the two-step feature strategy, the performances of the different feature selection strategies were compared and are listed in Table 1. The results show that the accuracy of the optimized multi-view features obtained by LightGBM-IFS outperformed the other feature selection methods for both full transcript and mature mRNA modes, with accuracies of 88.740% and 94.309% for tenfold CV, respectively. In addition, for full transcript mode, only the Sn value was lower than the ANOVA and *F*-score, with differences of only 0.782% and 0.521%, respectively, and the AUC, Sp, MCC, and F1 were 0.9504, 93.117%, 0.7493, and 0.8332, which were better than other feature selection methods. Similarly, for mature mRNA mode, the AUC, Sn, Sp, MCC, and F1 were 0.9792, 93.286%, 95.330%, 0.8864, and 0.9425, respectively, which were all better than the other three feature selection strategies.

**Table 1:** Performance comparison of the different feature selection strategies

Mode	Feature selection	Acc (%)	AUC	Sn (%)	Sp (%)	MCC	F1
Full transcript	ANOVA-IFS	87.853	0.9422	**81.356**	91.335	0.7312	0.8237
	F-score-IFS	87.648	0.9421	81.095	91.160	0.7267	0.8209
	**LightGBM -IFS**	**88.740**	**0.9504**	80.574	**93.117**	**0.7493**	**0.8332**
	XGBoost-IFS	88.376	0.9461	80.117	92.802	0.7412	0.8279
Mature mRNA	ANOVA-IFS	93.699	0.9756	92.879	94.518	0.8741	0.9364
	F-score-IFS	93.699	0.9756	92.879	94.518	0.8741	0.9363
	**LightGBM -IFS**	**94.309**	**0.9792**	**93.286**	**95.330**	**0.8864**	**0.9425**
	XGBoost-IFS	93.902	0.9735	92.777	95.025	0.8783	0.9383

**Fig. 4:**
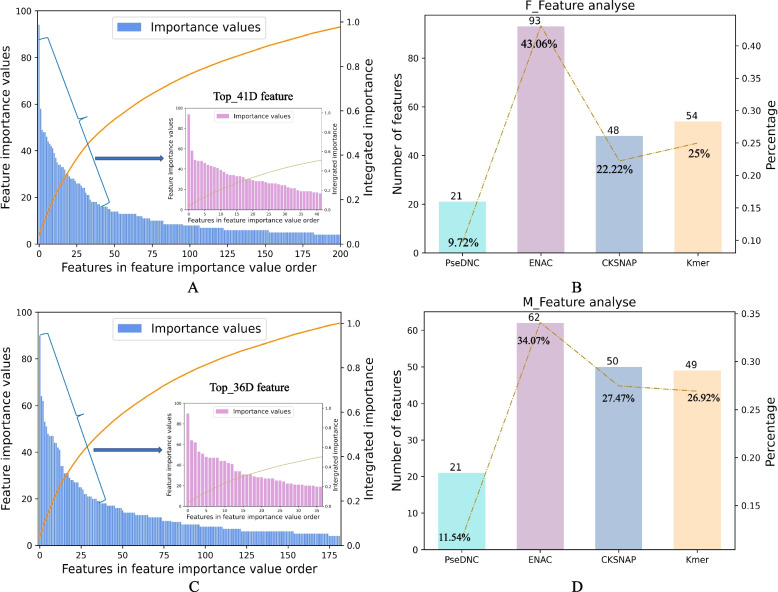
Four physical–chemical hybrid optimization feature analyses

The optimized features were obtained by feature selection and subjected to feature analysis. The results are listed in Fig. 4. For the m5U modification sites for full-transcript mode, a subset of 216D optimal features was selected from the four physicochemical multi-view features (497D) after employing a feature selection strategy combining LightGBM and IFS, and the features were ranked according to the feature importance values, as shown in Fig. 4A. The details of each of the top-ranked 41D optimized features are shown (Additional file 2: Table S3). The percentages of the four physicochemical features in the optimized feature subset and the number of all features for each feature are shown in Fig. 4B. Among them, the number of ENAC features was 93D, accounting for 43.06%, which is the largest share of the four physicochemical features, while the shares of PseDNC (21D), CKSNAP (48D), and Kmer (54D) were 9.72%, 22.22%, and 25.00%, respectively. Although the PseDNC feature was only 21D, it accounted for a higher proportion of the top 41D, and the feature importance value was relatively high. For the mature mRNA mode, similar to the m5U modification sites of the full transcript mode, a two-step feature selection strategy combining LightGBM and IFS was adopted to select a subset of 182D optimized features from the fused features of four physicochemical features. The features were ranked according to their importance values, as shown in Fig. 4C, where the top 36D optimal features contained the specific feature information shown in Additional file 2: Table S3. Similarly, as shown in Fig. 4D, for the identification of m5U modification sites of mature mRNA mode, ENAC was the feature with the highest number of feature dimensions among the four physicochemical features, with 62D features accounting for 34.07%, PseDNC (21D), CKSNAP (50D), and Kmer (49D), accounting for 11.54%, 27.47%, and 26.92%.

### Performance comparison of different machine learning algorithms

**Fig. 5:**
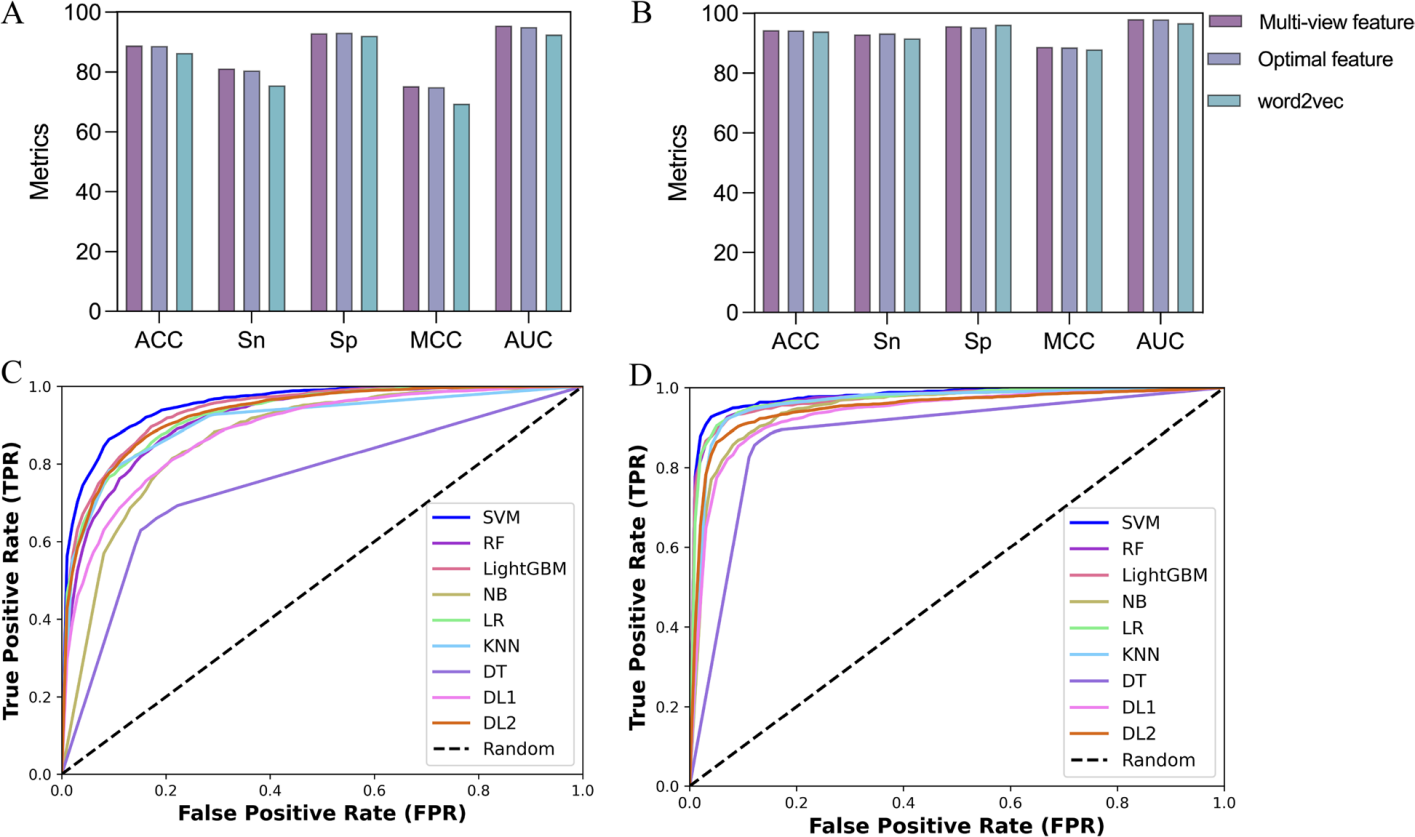
**A**, **B** Performance comparison of different feature representations for full transcript and mature mRNA modes under tenfold CV, respectively. **C**, **D** Performance comparison of different machine learning algorithms for full transcript and mature mRNA modes under tenfold CV, respectively

Optimized traditional multi-view physicochemical features of 216 and 182 dimensions were obtained for the m5U modification site sequences of full transcript and mature mRNA modes, respectively, under the LightGBM-IFS feature selection strategy. In addition to the traditional physicochemical features, we also used a distributed representation (word2vec) to encode the modification site sequence information. The word2vec method divides all trained modified nucleotide sequences into a Kmer corpus and then uses the skip-gram model to obtain 100-dimensional feature vectors for each Kmer. In this encoding process, the two optimized hyperparameters were the length $k$ of the nucleotide sequence Kmer (the length of the sliding window), and the window size $w$ (the number of surrounding words). The values of $k$ ranged from 2 to 10, and the values of $w$ ranged from 1 to 7. The two hyperparameters were combined, and the parameter-optimal skip-gram model was determined by comparing Acc. The features obtained by distributed representation were fused with optimized traditional features to obtain multi-view features for RNA m5U modification site prediction. For different modes of modification sites sequence, the performances of the optimized physicochemical features, the distributed representation features, and the multi-view features are compared in Fig. 5. The experimental results suggest that the multi-view features outperformed the single-view features and the optimized traditional physicochemical features based on tenfold CV. In Fig. 5A and B, the performance evaluation metrics of Acc, Sp, MCC, and AUC for the multi-view features were superior to the traditional physicochemical and distributed representation features. This shows that the multi-view fusion of optimized traditional physicochemical features with distributed representation features helps to improve the prediction accuracy of m5U modification sites.

The multi-view features of physicochemical and embedded features were integrated and screened after determining the optimal multi-view feature encoding for the modification site sequence. Next, we compare the results of different popular machine learning algorithms, including traditional machine learning methods and deep learning algorithms. Traditional machine learning methods include random forests (RF), SVM, logistic regression (LR), light gradient boosting machines (LightGBM), decision trees (DT), k-nearest neighbors (KNN), and naive Bayes (NB). The two deep learning architectures used for comparison are also commonly used in biological sequence analysis and prediction. The detailed architecture of the two models (DL1 and DL2) is introduced in Additional file 4: Figure S1. Briefly, DL1 is the hybrid of convolutional neural network (CNN) and long short-term memory (LSTM) and DL2 is the hybrid of CNN and gate recurrent unit (GRU). The hyperparameters for these machine learning algorithms are summarized in Additional file 3: Table S4. The comparative results of the tenfold CV and independent testing of these models are shown in Table 2, Fig. 5C and D. For full transcript modes, the SVM model achieved better performance than other machine learning algorithms. Among the five metrics, except for Sn, which was lower than DL1 and DL2, the Acc, Sp, MCC, and F1 were 88.876%, 92.977%, 0.7527, and 0.8360, respectively. The SVM also obtained the best AUC value of 0.9553. In addition, the Acc and AUC of SVM compared with the deep neural network approach were also superior to those of the deep learning algorithm, with Acc values higher than 8.47% and 4.993%, respectively, and similarly, AUCs higher than 7.103% and 2.525%, respectively. For the identification of mature mRNA mode m5U modification sites, the SVM algorithm outperformed other classification algorithms. The evaluation metrics of the SVM prediction model, except for Sn, were superior to those of the other algorithms. The obtained Acc, Sp, MCC, and F1 were 94.358%, 95.736%, 0.8875, and 0.9402, respectively. Similarly, under the independent test set, SVM outperformed other machine learning algorithms, with prediction accuracies of 90.821% and 94.106% for full transcript and mature mRNA modes, respectively. For different types of m5U modified sites, SVM was the best performing classifier under the tenfold CV and independent test set. Therefore, the SVM algorithm was chosen to construct the final predictive model.

**Table 2:** Performance comparison of different machine learning methods

Mode	Classifier	tenfold CV						Independent testing			
		**Acc (%)**	**Sn (%)**	**Sp (%)**	**MCC**	**F1**	**Acc (%)**	**Sn (%)**	**Sp (%)**	**MCC**	**F1**
Full transcript	**SVM**	**88.876**	81.226	**92.977**	**0.7527**	**0.8360**	**90.821**	**87.400**	**93.160**	**0.8091**	**0.8855**
	RF	83.963	68.188	92.418	0.6384	0.7479	86.758	78.000	92.749	0.7239	0.8271
	LightGBM	86.669	76.466	92.138	0.7022	0.8001	89.439	84.000	93.159	0.7800	0.8659
	NB	79.731	81.029	79.035	0.5802	0.7362	80.909	82.400	79.891	0.6144	0.7781
	LR	85.873	76.662	90.810	0.6854	0.7911	86.758	80.600	90.971	0.7237	0.8317
	KNN	86.510	78.292	90.915	0.7003	0.8020	88.789	84.800	91.518	0.7667	0.860
	DT	76.410	65.710	82.145	0.4797	0.6565	80.016	76.000	82.763	0.5865	0.7554
	DL1	80.406	89.891	66.540	0.5906	0.7323	80.259	71.800	86.046	0.5869	0.7471
	DL2	83.883	**90.684**	73.940	0.6712	0.7827	84.971	82.000	87.004	0.6889	0.8159
Mature mRNA	**SVM**	**94.358**	92.981	**95.736**	**0.8875**	**0.9402**	**94.106**	**93.061**	95.142	**0.8823**	**0.9402**
	RF	92.429	90.234	94.619	0.8494	0.9163	91.869	89.387	94.331	0.8383	0.9163
	LightGBM	92.378	90.946	93.807	0.8479	0.9215	92.276	91.020	93.522	0.8457	0.9214
	NB	88.161	91.150	85.178	0.7646	0.8706	86.585	90.612	82.591	0.7342	0.8705
	LR	92.632	92.472	92.792	0.8527	0.9084	90.853	91.020	90.688	0.8171	0.9083
	KNN	91.514	**94.608**	88.426	0.8319	0.9040	90.243	92.244	88.259	0.8055	0.9040
	DT	86.280	86.673	85.888	0.7256	0.8775	87.804	87.755	87.854	0.7561	0.8775
	DL1	87.947	91.094	84.775	0.7629	0.8750	88.008	83.670	92.307	0.7638	0.8742
	DL2	89.858	85.632	94.049	0.8021	0.8934	91.260	86.530	**95.951**	0.8295	0.9079

### Model interpretation

The interpretation of the model involves understanding how the model makes predictions and which features are most important in making predictions. Here, we evaluate the prediction process of a new model developed to understand the contribution of the corresponding features to the prediction model and to determine the impact of high or low feature importance scores on the prediction model. Recently, Lundberg et al. proposed the local interpretation method SHAP [37], which extends the concept of the Shapley value from game theory to introduce a uniform measure of feature importance. Machines can learn SHAP values to quantify the contribution of each feature in a predictive model [38]. SHAP values are output as an importance value ${\varnothing }_{i}\left(val\right)$ for each feature, indicating the effect of including that feature in the model training. Thus, for a given feature $i$, the SHAP value needs to be calculated for all possible combinations of features (including different orders) and then weighted to sum as follows:

13 $${\varnothing }_{i}\left(val\right)=\sum\nolimits_{k\subseteq \left\{{x}_{1},\dots ,{x}_{p}\right\}\setminus \left\{{x}_{i}\right\}}\frac{\left|k\right|!(p-\left|k\right|-1)!}{p!}\left(val\left(k\cup \left\{{x}_{i}\right\}\right)-val\left(k\right)\right)$$

where $p$ denotes the number of features, $x$ denotes a vector of eigenvalues of the samples to be interpreted, $k$ denotes a subset of the features used in the model, and $val\left(k\right)$ refers to the model output value under the feature combination $k$. To explain the weights, there are a total of $p$ features. Then, there are a total of $p!$ combinations of these $p$ features under the consideration of order, and after fixing a certain feature $i$, the remaining ones have $(p-\left|k\right|-1)!k!$ combinations. The greatest advantage of the SHAP value is that it can reflect the influence of the features in each sample and shows positive and negative influences.

**Fig. 6:**
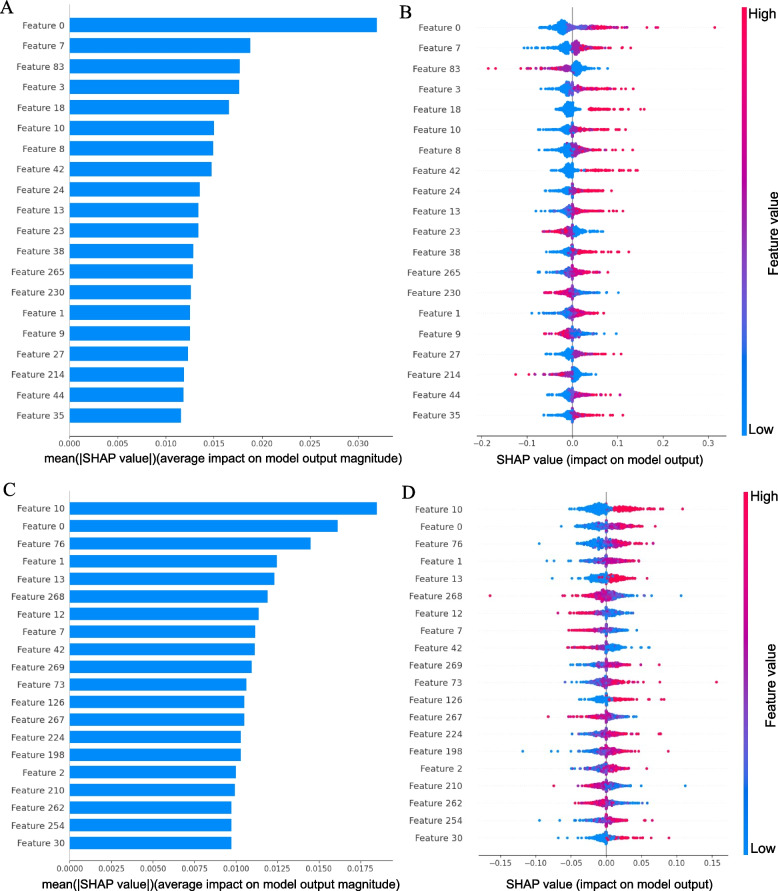
**A**, **C** The 20 most important features. **B**, **D** The top 20 important features are sorted by SHAP value, and the impact of each feature value is displayed

Which features are most important to the prediction model is determined by calculating feature importance values using SHAP, and the results are displayed in Fig. 6 and Additional file 3: Table S5. For full transcript and mature mRNA modes, SHAP summary plots were used to calculate the top 20 important feature rankings, and the feature importance distribution was obtained by taking the absolute average of the SHAP values for each feature. The results are shown in Fig. 6A and C, and the top 20 important features are presented in detail in the Additional file 3: Table S5. For the full transcript mode m5U modification sites, the top 20 features contained 18 physicochemical features and 2 embedded features; similarly, for the mature mRNA mode m5U modification sites, the top 20 important features contained 12 physicochemical features and 8 embedded features. This indicates that both types of features are important for the construction of predictive models. Among them, the PseDNC_7 feature was in the first and second positions among the top 20 features for full transcript and mature mRNA modes, respectively, indicating that this feature contributed more to the model prediction. In addition, Fig. 6B and D show that when observing the importance of the features, there is an intuitive judgment on how the features affect the overall predicted value, that is, sorted by the sum of the SHAP value sizes of the samples, the distribution of the effect of each feature on the prediction model output can be illustrated to determine which features are most important for predicting the model. Each point in the honeycomb plot represents a real sample. For each group (each row), the color of the data points is determined by the value of the feature. The larger the value of the feature is, the redder the color of the point. The more points there are with the same SHAP value, the larger the cross-sectional area of the “honeycomb” and the thicker it will appear. For example, for the full transcript mode, the PseDNC_7 feature has the greatest effect on the model; when the value of PseDNC_7 is smaller, the output of the model is smaller, and vice versa, the larger the value is, the larger the output of the model. Similarly, for mature mRNA mode, the PseDNC_18 feature has the greatest effect on the model, and the value is positively correlated with the model output size. When the value of PseDNC_18 is smaller, the output of the model is smaller, and vice versa, the larger the value is, the larger the output of the model. To understand how a single feature affects the output of the model, we show the impact of individual features on the model output using SHAP dependency plots for the top 20 features (refer to Additional file 4: Figures S2 and S3).

### Comparison with the published method

m5UPred [17] is a tool developed to predict RNA m5U modification sites. Here, we used data derived from m5UPred using CD-HIT (threshold of 0.8) to remove homologous sequences. Therefore, to compare the performance of the developed new method with the m5UPred, we used the m5UPred to perform predictions on the processed data. The results of the performance comparison between the developed new method for m5U identification and the m5UPred are shown in Table 3. The prediction performance of our developed new predictor was better than that of the m5UPred. For full transcript mode m5U modification sites, the performance of the model was evaluated under tenfold CV, and the evaluation indicators obtained by the newly developed method for Acc, AUC, Sn, Sp, and MCC were 88.876%, 95.527%, 81.226%, 92.977%, and 0.7527, respectively. In terms of accuracy, m5U-SVM improved over the m5UPred by 5.278%. Similarly, for mature mRNA mode m5U modification sites, the evaluation metrics of the newly developed method for Acc, AUC, Sn, Sp, and MCC were 94.358%, 98.038%, 92.981%, 95.736%, and 0.8875, respectively. The performance metrics of the newly developed predictor were superior, and the accuracy was improved by 4.448% compared with the m5UPred. Under the independent testing, the newly developed predictor is better than the m5UPred for the prediction of modification sites in the full transcript and mature mRNA modes, with an accuracy improvement of 3.656% and 4.406%. The comparison of the experimental results suggests that the novel developed predictor is superior to the published method.

**Table 3:** Performance comparison with published methods

Mode	Method	Cross validation					Independent testing				
		**Acc (%)**	**AUC**	**Sn (%)**	**Sp (%)**	**MCC**	**Acc (%)**	**AUC**	**Sn (%)**	**Sp (%)**	**MCC**
Full transcript	m5U-SVM	**88.876**	**0.9553**	**81.226**	**92.977**	**0.7527**	**90.821**	**0.9667**	**87.399**	**93.160**	**0.8092**
	m5UPred	83.598	0.9109	72.816	89.378	0.6338	87.165	0.9450	80.600	91.656	0.7322
Mature mRNA	m5U-SVM	**94.358**	**0.9804**	**92.981**	**95.736**	**0.8875**	**94.106**	**0.9621**	**93.061**	**95.142**	**0.8823**
	m5UPred	89.910	0.9560	88.640	91.180	0.7980	89.700	0.9540	87.440	91.950	0.7950

## Conclusions

RNA 5-methyluridine modification sites play crucial roles in biological processes as one of the posttranscriptional modifications that occurs mainly as a methylation substitution at the C_5_ position of uridine. To investigate the biological functions of the modification sites in greater depth, it is necessary to develop highly accurate tools for m5U site prediction. Therefore, we proposed a novel prediction tool, m5U-SVM, for m5U modification site prediction, which is a model constructed based on multi-view features with SVM. The multi-view features mainly contain distributed representation and splicing of physicochemical features. Two-step feature selection is used to optimize the spliced multi-view features and obtain the important features that can identify m5U modification sites. Experimental studies based on cross-validation demonstrate the validity and robustness of the m5U-SVM model in comparison with existing methods. For full transcript and mature mRNA modes, the accuracies of the tenfold CV obtained by our developed method were 88.876% and 94.358%, and the accuracies of the independent testing were 90.821% and 94.106%. In addition, we employed SHAP to investigate the most important features for predicting m5U modification sites, and the features were analyzed.

Although the current prediction method for identifying m5U-modified sites and non-m5U-modified sites works well, there are still some limitations. First, the dataset can be expanded in the future with data and species types. Second, it would be interesting to incorporate sequence-based chemical structures and more applied deep learning methods into the framework of the proposed method to improve the prediction performance of the model in future studies.

## Supplementary Information

**Additional file 1:** **Table S1.** Physicochemical property descriptor parameter search range and the best values. **Table S2.** Performance comparison under tenfold CV and independent test set for different feature descriptors.

**Additional file 2:** **Table S3.** The detailed feature types contained in the optimal feature subsets of full transcript and mature mRNA modes.

**Additional file 3:** **Table S4.** Hyperparameters for different classification algorithms. **Table S5.** The SHAP summary plot was used to calculate the top 20 most important feature details.

**Additional file 4:** **Figure S1.** The structure of the deep neural network framework, including the input layer, convolutional layers, merger layers, fully connected layers, and output layer. A and B represent DL1 and DL2 in the main text, respectively. **Figure S2.** The SHAP dependence plots for full transcript mode m5U sites. These plots illustrate the effect that a single feature has on the models predictions and the interaction effects across features. Each point represents an individual sample, with the value on the x-axis representing the value of the feature in question and the color indicating the value of the interacting feature. **Figure S3.** The SHAP dependence plots for mature mRNA mode m5U sites. These plots illustrate the effect that a single feature has on the models predictions and the interaction effects across features. Each point represents an individual sample, with the value on the x-axis representing the value of the feature in question and the color indicating the value of the interacting feature.

**Additional file 5.** Metrics for performance evaluation.

## Data Availability

The publicly available webserver is freely accessible at http://lab.malab.cn/~acy/m5USVM. The datasets of training and testing can be downloaded from https://github.com/aochunyan/m5U-SVM/tree/main/Dataset. The source code of m5U-SVM [39] are available at https://github.com/aochunyan/m5U-SVM.git. All data generated or analyzed during this study are included in this published article, its supplementary information files and publicly available repositories.

## Abbreviations

m5U: 5-Methyluridine
Acc: Accuracy
ANOVA: Analysis of variance
CKSNAP: Composition of k-spaced nucleic acid pairs
CBOW: Continuous bag-of-words
CNN: Convolutional neural network
DT: Decision trees
ENAC: Enhanced nucleic acid composition
F1: F1-score
GRU: Gate recurrent unit
IFS: Incremental feature selection
KNN: K-nearest neighbors
LightGBM: Light gradient boosting machines
LR: Logistic regression
LSTM: Long short-term memory
MCC: Matthew’s correlation coefficient
NAC: Nucleic acid composition
NB: Naive Bayes
PseDNC: Pseudo dinucleotide composition
PseNAC: Pseudo nucleic acid composition
RF: Random forests
SVM: Support vector machine
Sn: Sensitivity
Sp: Specificity
SHAP: Shapley Additive explanation
