# Supplementary material for: m5U-SVM: identification of RNA 5-methyluridine modification sites based on multi-view features of physicochemical features and distributed representation
Source: BMC Biol. 2023 Apr 24;21:93. doi: 10.1186/s12915-023-01596-0 (PMC10127088; doi:10.1186/s12915-023-01596-0)
Supplement: Supplementary file 1 — Additional file 1: Table S1. Physicochemical property descriptor parameter search range and the best values. Table S2. Performance comparison under tenfold CV and independent test set for different feature descriptors. [file 12915_2023_1596_MOESM1_ESM.docx]

For the optimization of the parameters of the four physicochemical property feature descriptors, with the full transcript training dataset, the random forest (RF) algorithm was employed for evaluation based on 10-fold cross-validation. The results of the optimized parameters for different physicochemical characteristics are shown in Table S1.

**Table S1.** Physicochemical property descriptor parameter search range and the best values.

| Feature encoding | Parameters range | Optimal parameters | Acc |
| --- | --- | --- | --- |
| ENAC | Sliding window size [2, …,6] | 3 | 83.62% |
| Kmer | k [1,2,3,4,5] | 4 | 83.78% |
| PseDNC | lamada(λ) [1, 2, 3, ……,10] | 5 | 80.755% |
| CKSNAP | gap(k) [0,1,2,3,4,5] | 3 | 81.755% |

**Table S2**. Performance comparison under 10-fold CV and independent test set for different feature descriptors.

| Mode | Feature encoding | 10-fold CV | | | | | | Independent testing | | | | | |
| --- | --- | --- | --- | --- | --- | --- | --- | --- | --- | --- | --- | --- | --- |
|  |  | Acc (%) | Sn (%) | Sp (%) | MCC | Precision (%) | F1 | Acc (%) | Sn (%) | Sp (%) | MCC | Precision (%) | F1 |
| Full transcript | ENAC (1) | 84.554 | 75.614 | 89.343 | 0.6574 | 79.257 | 0.7731 | 83.992 | 77.200 | 88.646 | 0.6681 | 79.990 | 0.7813 |
|  | Kmer (2) | 84.507 | 72.411 | 90.988 | 0.6528 | 81.148 | 0.7643 | 82.772 | 76.800 | 86.867 | 0.6426 | 82.735 | 0.7720 |
|  | PseDNC (3) | 81.072 | 65.239 | 89.554 | 0.5725 | 76.971 | 0.7046 | 82.935 | 73.200 | 89.605 | 0.6446 | 80.893 | 0.7637 |
|  | CKSNAP (4) | 82.304 | 69.025 | 89.414 | 0.6027 | 77.771 | 0.7302 | 82.205 | 73.000 | 88.511 | 0.6278 | 82.387 | 0.7936 |
|  | 1+2+3+4 | **88.217** | **79.987** | **92.628** | **0.7377** | **85.327** | **0.8257** | **90.1706** | **86.200** | **92.886** | **0.7955** | **89.234** | **0.8769** |
| Mature mRNA | ENAC (1) | 90.398 | 88.302 | 92.494 | 0.8095 | 94.300 | 0.9310 | 85.947 | 84.750 | 87.017 | 0.7258 | 90.712 | 0.8778 |
|  | Kmer (2) | 92.123 | 91.042 | 93.199 | 0.8428 | 92.277 | 0.9020 | 87.573 | 82.183 | 92.683 | 0.7639 | 86.896 | 0.8500 |
|  | PseDNC (3) | 89.123 | 90.435 | 87.804 | 0.7833 | 93.063 | 0.9203 | 85.156 | 85.900 | 84.184 | 0.7122 | 91.734 | 0.8508 |
|  | CKSNAP (4) | 93.190 | 91.964 | 94.417 | 0.8643 | 88.204 | 0.8927 | 89.214 | 86.767 | 91.467 | 0.7912 | 83.992 | 0.8412 |
|  | 1+2+3+4 | **94.106** | **92.9807** | **95.228** | **0.8823** | **95.109** | **0.9403** | **93.089** | **92.653** | **93.522** | **0.8618** | **93.416** | **0.9303** |

Note: Bold indicates best results.
