# Supplementary material for: m5U-SVM: identification of RNA 5-methyluridine modification sites based on multi-view features of physicochemical features and distributed representation
Source: BMC Biol. 2023 Apr 24;21:93. doi: 10.1186/s12915-023-01596-0 (PMC10127088; doi:10.1186/s12915-023-01596-0)
Supplement: Supplementary file 2 — Additional file 2: Table S3. The detailed feature types contained in the optimal feature subsets of full transcript and mature mRNA modes. [file 12915_2023_1596_MOESM2_ESM.docx]

**Table S3**. The detailed feature types contained in the optimal feature subsets of full transcript and mature mRNA modes.

| Full transcript mode | | Mature mRNA mode | |
| --- | --- | --- | --- |
| Top-41D (Optimal feature) | Feature importance value | Top-36D (Optimal feature) | Feature importance value |
| PseDNC_7 | 94 | PseDNC_7 | 90 |
| PseDNC_19 | 58 | CKSNAP_50 | 64 |
| PseDNC_18 | 49 | PseDNC_19 | 62 |
| ENAC_63 | 48 | PseDNC_11 | 53 |
| PseDNC_15 | 48 | PseDNC_16 | 51 |
| PseDNC_20 | 46 | PseDNC_12 | 48 |
| ENAC_110 | 44 | PseDNC_21 | 47 |
| ENAC_84 | 43 | PseDNC_15 | 47 |
| ENAC_73 | 42 | PseDNC_13 | 47 |
| CKSNAP_52 | 41 | PseDNC_18 | 44 |
| ENAC_90 | 39 | Kmer_247 | 44 |
| ENAC_94 | 37 | PseDNC_17 | 42 |
| PseDNC_2 | 35 | CKSNAP_32 | 41 |
| CKSNAP_50 | 34 | Kmer_176 | 34 |
| PseDNC_5 | 34 | PseDNC_14 | 34 |
| PseDNC_11 | 33 | ENAC_84 | 31 |
| PseDNC_6 | 33 | CKSNAP_44 | 31 |
| PseDNC_21 | 32 | PseDNC_20 | 31 |
| Kmer_217 | 30 | PseDNC_1 | 29 |
| CKSNAP_32 | 30 | PseDNC_6 | 28 |
| PseDNC_12 | 29 | PseDNC_8 | 28 |
| PseDNC_16 | 28 | PseDNC_5 | 27 |
| PseDNC_14 | 28 | PseDNC_3 | 27 |
| ENAC_109 | 28 | PseDNC_4 | 27 |
| ENAC_19 | 27 | Kmer_14 | 25 |
| ENAC_47 | 26 | CKSNAP_43 | 25 |
| PseDNC_17 | 26 | CKSNAP_28 | 24 |
| ENAC_77 | 26 | CKSNAP_59 | 22 |
| PseDNC_3 | 25 | ENAC_102 | 22 |
| PseDNC_1 | 24 | PseDNC_2 | 21 |
| PseDNC_8 | 24 | Kmer_209 | 21 |
| ENAC_55 | 22 | PseDNC_9 | 21 |
| PseDNC_9 | 21 | CKSNAP_64 | 20 |
| PseDNC_10 | 21 | CKSNAP_42 | 20 |
| ENAC_43 | 19 | CKSNAP_27 | 20 |
| ENAC_123 | 18 | ENAC_24 | 19 |
| CKSNAP_23 | 18 | / | / |
| CKSNAP_62 | 18 | / | / |
| ENAC_114 | 18 | / | / |
| CKSNAP_33 | 17 | / | / |
| PseDNC_13 | 17 | / | / |
