# Supplementary material for: m5U-SVM: identification of RNA 5-methyluridine modification sites based on multi-view features of physicochemical features and distributed representation
Source: BMC Biol. 2023 Apr 24;21:93. doi: 10.1186/s12915-023-01596-0 (PMC10127088; doi:10.1186/s12915-023-01596-0)
Supplement: Supplementary file 3 — Additional file 3: Table S4. Hyperparameters for different classification algorithms. Table S5. The SHAP summary plot was used to calculate the top 20 most important feature details. [file 12915_2023_1596_MOESM3_ESM.docx]

**Table S4.** Hyperparameters for different classification algorithms.

| **Method** | **Parameter** | **Tested values** |
| --- | --- | --- |
| LR | C | 0.1-5 with an interval of 1 |
| SVM | C | [2^-5^–2^15^] in log_2_ steps |
|  | gamma | [2^-15^–2^-5^] in log_2_ steps |
|  | kernel | RBF |
| DT | max_features | 0-11with an interval of 1 |
|  | min_samples_split | 1-11with an interval of 1 |
| RF | n_estimators | 10-200 with an interval of 20 |
|  | max_features | 1-10 with an interval of 2 |
| KNN | n_neighbors | 1-100 with an interval of 1 |
| LightGBM | n_estimators | 10-200 with an interval of 20 |
|  | learning_rate | 0.01-0.1 with an interval of 0.01 |
| DL1/DL2 | learning_rate | [0.0001, 0.001] |
|  | weight decay | [0.00004, 0.0002] |
|  | batch_size | 128 |
|  | conv1_filter | 32 |
|  | conv2_filter | 32 |
|  | dropoutMerge1 | 0.5 |
|  | dropoutMerge2 | 0.5 |
|  | AdaptiveAvgPool1d | 128 |
|  | dense | 64 |

**Table S5**. The SHAP summary plot was used to calculate the top 20 most important feature details.

| Full transcript mode | Mature mRNA mode |
| --- | --- |
| PseDNC_7 | Kmer_247 |
| ENAC_84 | PseDNC_7 |
| ENAC_78 | CKSNAP_7 |
| ENAC_63 | CKSNAP_50 |
| Kmer_217 | Kmer_176 |
| ENAC_90 | word2vec_87 |
| ENAC_73 | CKSNAP_32 |
| Kmer_91 | PseDNC_15 |
| ENAC_19 | ENAC_83 |
| CKSNAP_50 | word2vec_88 |
| ENAC_109 | CKSNAP_34 |
| ENAC_114 | Kmer_54 |
| word2vec_50 | word2vec_86 |
| word2vec_15 | word2vec_43 |
| PseDNC_19 | word2vec_17 |
| CKSNAP_52 | PseDNC_19 |
| ENAC_77 | word2vec_29 |
| ENAC_75 | word2vec_81 |
| ENAC_67 | word2vec_73 |
| ENAC_123 | Kmer_209 |
