# Supplementary material for: m5U-SVM: identification of RNA 5-methyluridine modification sites based on multi-view features of physicochemical features and distributed representation
Source: BMC Biol. 2023 Apr 24;21:93. doi: 10.1186/s12915-023-01596-0 (PMC10127088; doi:10.1186/s12915-023-01596-0)
Supplement: Supplementary file 4 — Additional file 4: Figure S1. The structure of the deep neural network framework, including the input layer, convolutional layers, merger layers, fully connected layers, and output layer. A and B represent DL1 and DL2 in the main text, respectively. Figure S2. The SHAP dependence plots for full transcript mode m5U sites. These plots illustrate the effect that a single feature has on the models predictions and the interaction effects across features. Each point represents an individual sample, with the value on the x-axis representing the value of the feature in question and the color indicating the value of the interacting feature. Figure S3. The SHAP dependence plots for mature mRNA mode m5U sites. These plots illustrate the effect that a single feature has on the models predictions and the interaction effects across features. Each point represents an individual sample, with the value on the x-axis representing the value of the feature in question and the color indicating the value of the interacting feature. [file 12915_2023_1596_MOESM4_ESM.docx]

**Figure S1.** The structure of the deep neural network framework, including the input layer, convolutional layers, merger layers, recurrent layers, fully connected layers, and output layer. A and B represent DL1 and DL2 in the main text, respectively.

**
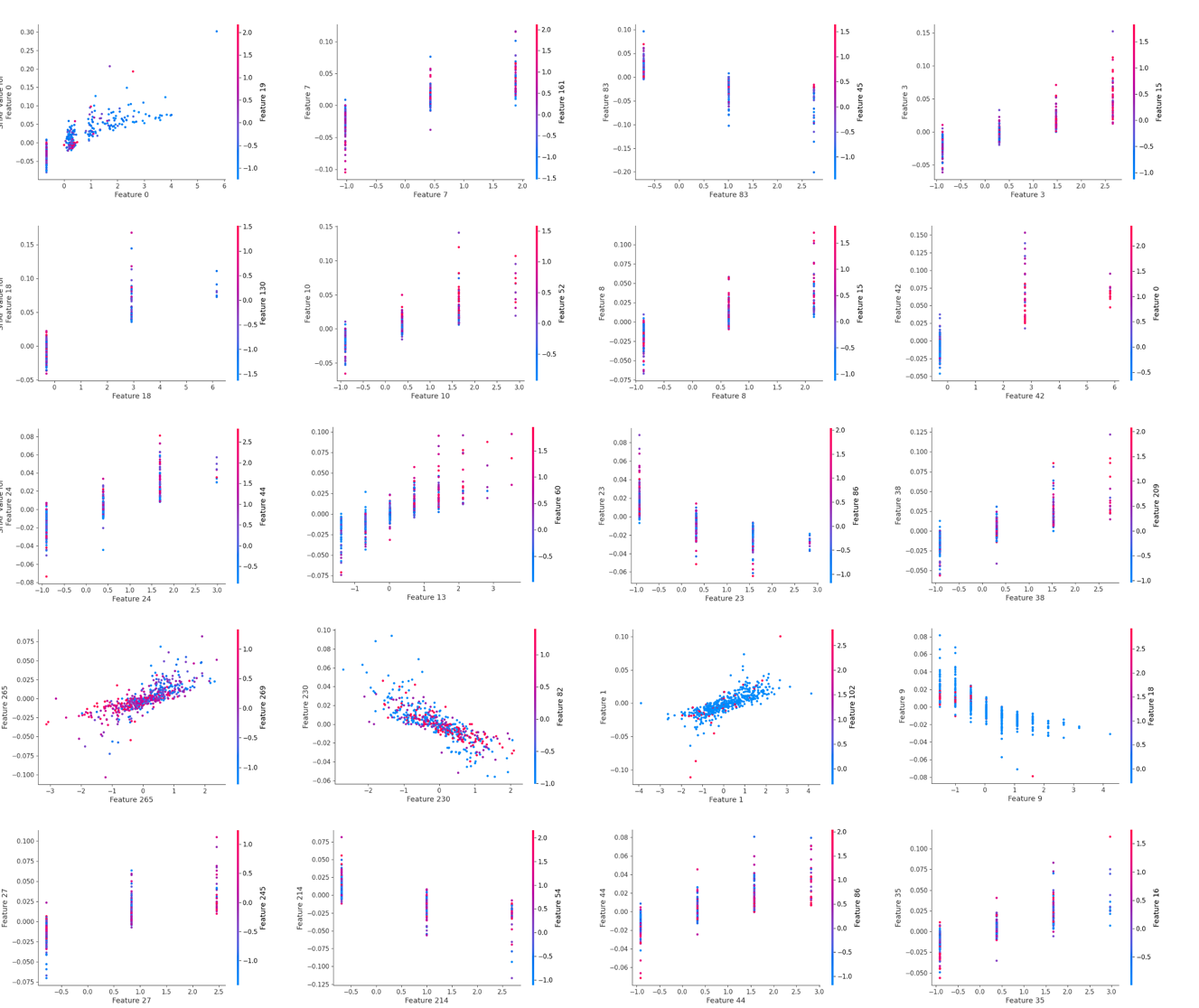
**

**F****igure S2.** The SHAP dependence plots for full transcript mode m5U sites. These plots illustrate the effect that a single feature has on the models predictions and the interaction effects across features. Each point represents an individual sample, with the value on the x-axis representing the value of the feature in question and the color indicating the value of the interacting feature.


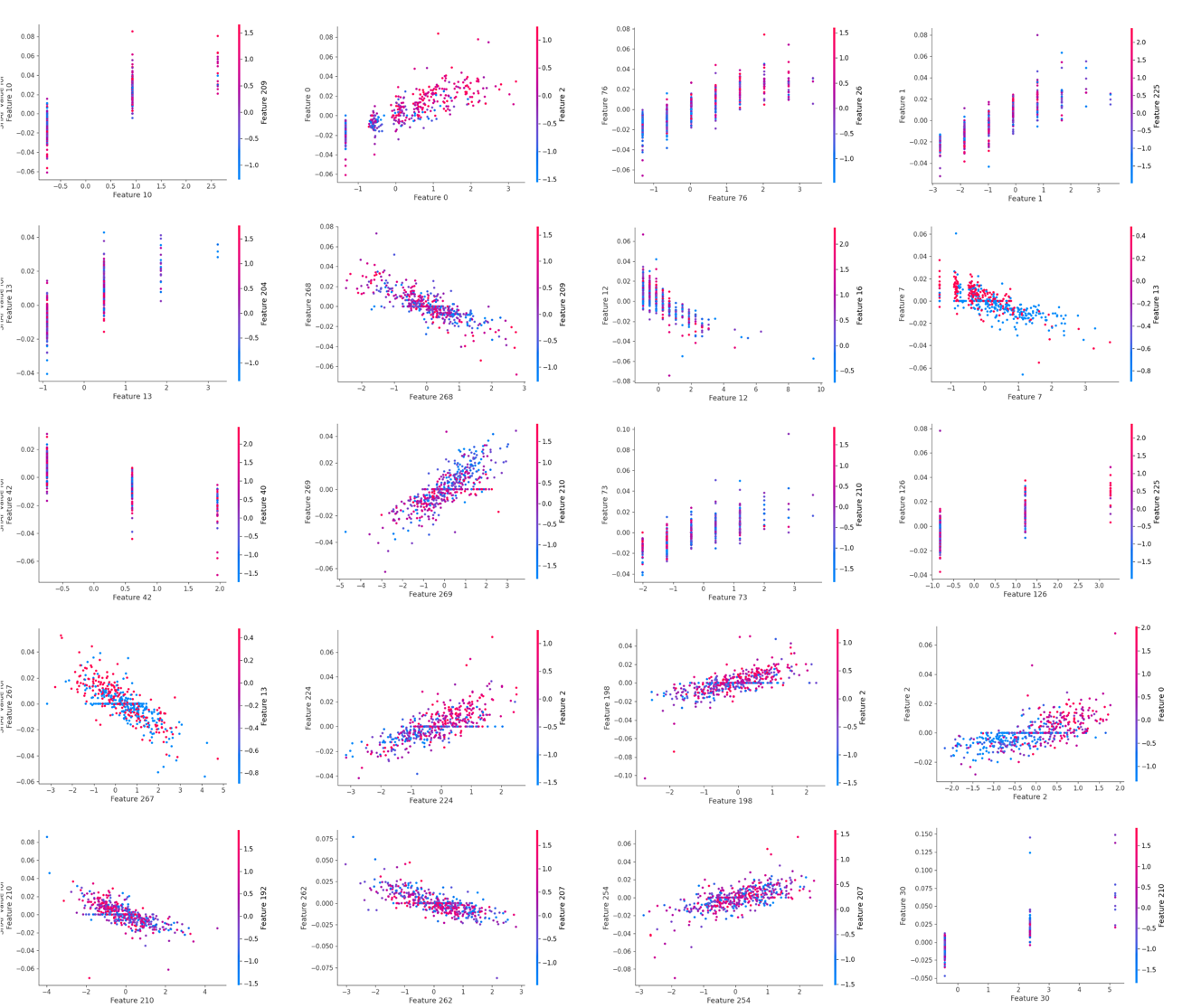


**Figure S3.** The SHAP dependence plots for mature mRNA mode m5U sites. These plots illustrate the effect that a single feature has on the models predictions and the interaction effects across features. Each point represents an individual sample, with the value on the x-axis representing the value of the feature in question and the color indicating the value of the interacting feature.
