# Supplementary material for: m5U-SVM: identification of RNA 5-methyluridine modification sites based on multi-view features of physicochemical features and distributed representation
Source: BMC Biol. 2023 Apr 24;21:93. doi: 10.1186/s12915-023-01596-0 (PMC10127088; doi:10.1186/s12915-023-01596-0)
Supplement: Supplementary file 5 — Additional file 5. Metrics for performance evaluation. [file 12915_2023_1596_MOESM5_ESM.docx]

1. **Metrics for performance evaluation**

We used several general performance indicators, including Sensitivity (Sn), Specificity (Sp), Accuracy (Acc), Precision (PRE), Mathew’s correlation coefficient (MCC) and F1-score (F1). They are computed as follows:

$S_{p}=\frac{TN}{TN+FP}$ (1)

$S_{n}=\frac{TP}{TP+FN}$ (2)

$Acc=\frac{TP+TN}{TP+TN+FP+FN}$ (3)

$Precision=\frac{TP}{TP+FP}$ (4)

$MCC=\frac{TP\times TN-FP\times FN}{\sqrt{(TP+FP)\times(TP+FN)\times(TN+FN)\times(TN+FP)}}$ (5)

$F1=\frac{2\times TP}{2\times TP+FP+FN}$ (6)

where TP, FP, TN, and FN represent the number of true positive, false positive, true negative and false negative samples in the prediction of m5U modification sites, respectively. In addition, we calculated AUC (the area under the receiver operating characteristic (ROC) curve) and AUPR (the area under the PR curve) of the two indices as comprehensive evaluation indexes.
